# Significance and Determinants of Plasma Apelin in Patients With Obstructive Hypertrophic Cardiomyopathy

**DOI:** 10.3389/fcvm.2022.904892

**Published:** 2022-06-17

**Authors:** Chengzhi Yang, Changlin Zhang, Ruofei Jia, Shubin Qiao, Jiansong Yuan, Zening Jin

**Affiliations:** ^1^Department of Cardiology and Macrovascular Diseases, Beijing Tiantan Hospital, Capital Medical University, Beijing, China; ^2^Department of Cardiology, The Second Affiliated Hospital of Dalian Medical University, Dalian, China; ^3^Department of Cardiology, National Center for Cardiovascular Diseases, Fuwai Hospital, Chinese Academy of Medical Sciences and Peking Union Medical College, Beijing, China

**Keywords:** apelin, obstructive hypertrophic cardiomyopathy, septal wall thickness, myocardial hypertrophy, right ventricular end-diastolic diameter

## Abstract

**Background:**

Recent studies suggest apelin has multiple protective effects in some cardiovascular diseases. However, there are few data concerning apelin levels in patients with obstructive hypertrophic cardiomyopathy (OHCM) or the relationship between apelin levels and severity of OHCM.

**Methods:**

We studied 88 patients with OHCM and 32 control subjects with matched age and sex distribution. Complete medical history was collected and related examinations were performed. Cardiac magnetic resonance (CMR) and echocardiography were employed to characterize cardiac morphology and function. Plasma apelin was measured by enzyme-linked immunosorbent assay (ELISA).

**Results:**

Plasma apelin levels were significantly lower in patients with OHCM than those in control subjects (96.6 ± 34.3 vs. 169.4 ± 62.5 μg/L, *p* < 0.001). When patients with OHCM were divided into two groups according to the mean value of plasma apelin, patients with lower apelin levels (plasma apelin ≤ 96.6 μg/L) had greater septal wall thickness (SWT; 25.6 ± 5.5 vs. 23.2 ± 4.3 mm, *p* = 0.035) and less right ventricular end-diastolic diameter (RVEDD; 20.4 ± 3.3 vs. 23.0 ± 3.6 mm, *p* = 0.001). Consistently, plasma apelin levels were inversely correlated with SWT (*r* = −0.334, *p* = 0.002) and positively correlated with RVEDD (*r* = 0.368, *p* < 0.001). Besides, plasma apelin levels were inversely correlated with Ln (NT-proBNP) (*r* = −0.307, *p* = 0.008) and positively correlated with body mass index (BMI; *r* = 0.287, *p* = 0.008). On multivariate analysis, the SWT was independently associated with decreasing plasma apelin, while the RVEDD was independently associated with increasing plasma apelin.

**Conclusion:**

Plasma apelin levels are reduced in patients with OHCM. The apelin levels are inversely related to SWT and positively related to RVEDD.

## Introduction

Hypertrophic cardiomyopathy (HCM) is a primary inherited myocardial disorder characterized by asymmetrical left ventricular hypertrophy (LVH), with a prevalence of 1:500 in the general population (1). Notably, about 70% of patients with HCM have left ventricular outflow tract (LVOT) obstruction, which is called obstructive hypertrophic cardiomyopathy (OHCM) (1). The presence of LVOT obstruction prominently aggravates clinical symptoms of HCM, such as dyspnea, chest pain and syncope. The LVOT obstruction is often attributed to septal hypertrophy and systolic anterior motion of the mitral valve, and the former is the pathological basis of OHCM (2). However, there are few findings concerning circulating biomarkers that can reflect the severity of LVH in OHCM.

Apelin is an endogenous peptide isolated from bovine stomach extracts (3). Recent studies have shown that apelin has diverse protective effects in many cardiovascular diseases, such as heart failure, systemic and pulmonary arterial hypertension and ischemic-reperfusion lesion (4, 5). Of note, Scimia et al. reported that apelin administration blunts progression to cardiac hypertrophy induced with transverse aortic constriction in mice (6). And our previous study found that apelin could inhibit angiotensin II-induced myocardial hypertrophy (7). These findings suggest that apelin may have some effects on LVH in patients with OHCM. Currently, however, there are few studies concerning the relationship between apelin levels and characteristics indicating severity of OHCM. In this study, we sought to investigate apelin levels in patients with OHCM compared with control subjects and their association with clinical and cardiac morphological characteristics of patients.

## Materials and Methods

### Study Population

The protocol of this study was approved by Fuwai Hospital (Beijing, China) ethics committee and complied with the Declaration of Helsinki. The informed consents were obtained from all participants.

We enrolled patients with OHCM who were evaluated in Fuwai Hospital (Beijing, China) from October 2015 to October 2016. The diagnosis of OHCM was based on a maximum left ventricular (LV) wall thickness ≥ 15 mm (or ≥ 13 mm with an unequivocal family history of HCM) and the presence of LVOT obstruction, as measured by echocardiography or cardiac magnetic resonance imaging (CMRI), in the absence of other cardiac or systemic diseases capable of producing comparable magnitude of hypertrophy (8). Evaluation of patients included complete medical history, physical examination, 12-lead electrocardiography, 24-h ambulatory electrocardiographic monitoring, transthoracic echocardiography, blood examination, CMRI, and coronary angiography.

Patients with coronary artery disease (epicardial coronary stenosis > 70% on coronary angiography, previous myocardial infarction, bypass surgery, or percutaneous coronary intervention), renal dysfunction, liver diseases, or permanent mechanical device implantation were excluded. Finally, a total of 88 patients with OHCM were recruited in the present study.

Thirty-two asymptomatic subjects with matched age and sex distribution of the OHCM patients were invited to participate as controls after detailed clinical and cardiac examination.

### Measurement of Plasma Apelin and N-Terminal pro-B-Type Natriuretic Peptide Levels

Fasting venous blood samples of patients with OHCM were collected in tubes containing EDTA within 2 days of echocardiography and 1 week of CMRI examination. Fasting blood samples of control subjects were collected at the same day of echocardiography examination. Blood samples were subsequently centrifuged for 15 min at 3,000 g. Then plasma was collected and stored at −80°C until assay. Plasma concentration of apelin was measured by a commercial enzyme-linked immunosorbent assay (ELISA) kit for human apelin (RayBiotech, Inc., Norcross, GA, United States) according to the manufacturer’s instructions. This kit is designed to target the C-terminus of the 77-aa apelin peptide and therefore is expected to detect all active forms of apelin, including apelin-36, apelin-31, apelin-28, and apelin 13. The intra-assay coefficient of variation was < 10% and the inter-assay coefficient of variation was < 15%. The lower and upper limit of detection was 0.1 and 1,000 μg apelin/L, respectively. NT-proBNP was measured using an electrochemiluminescent immunoassay (Elecsys proBNP II assay; Roche Diagnostics, Mannheim, Germany) by the clinical chemistry department of our hospital.

### Echocardiography

Standard transthoracic M-mode, 2-dimensional, and pulse-wave and continuous-wave Doppler images were obtained with an iE33 Color Doppler Ultrasound System (Philips Healthcare, Andover, Massachusetts). All measurements were analyzed following the guidelines of the American Society of Echocardiography (9). The peak velocity across the LVOT was measured and the peak pressure gradient was estimated using the simplified Bernouilli equation. The presence of LVOT obstruction was defined as an instantaneous peak Doppler LVOT gradient ≥ 30 mm Hg at rest or during physiological provocation, such as Valsalva maneuver, standing, and exercise.

### Cardiac Magnetic Resonance Imaging

CMRI was performed using a 1.5-T speed clinical scanner (Magnetom Avanto; Siemens Medical Solutions, Erlangen, Germany). The imaging protocol and analysis have been described previously (10). All MR image analysis was performed using a commercial software (Medis Medical Imaging systems, Netherlands) by a single experienced observer who was blinded to the patients’ clinical and procedural data. Endocardial and epicardial contours of the LV myocardium (excluding papillary muscles) were manually traced at end-diastole and end-systole on each LV short-axis cine image. LV end-diastolic volume, LV end-systolic volume, LVEF, stroke volume, cardiac output, and LV mass (LVM) were then calculated in a standard fashion. LVM was derived by multiplying LV myocardial volume measured at end-diastole with the specific gravity of myocardium (1.05 g/ml). The LV end-diastolic diameter, septal wall thickness and right ventricular end-diastolic diameter were traced and measured from the short-axis views at end-diastole.

### Statistical Analysis

Continuous variables are expressed as mean ± SD or median [interquartile range (IQR)], according to their normality. Categorical variables are shown as frequencies (percentages). Comparisons of continuous variables between two groups were assessed using independent Student’s *t*-test or Mann-Whitney *U*-test depending on the distribution of variables. The chi-square test was used for comparisons between categorical variables, and Fisher’s exact test was used when expected frequency was < 5. Pearson’s correlation test or Spearman’s correlation test was used to examine correlations between two continuous variables (as appropriate). Logarithmic transformations were performed for NT-proBNP to obtain normal distribution. Stepwise multiple linear regression analysis (*p*-value threshold to enter 0.05; to remove, 0.10) was conducted to identify independent variables that might determine plasma apelin levels. Variables with a *p*-value < 0.10 in the univariate analysis were included in the multiple regression analysis. A 2-tailed *p*-value < 0.05 was considered as statistically significant. Statistical analysis was performed with SPSS software (IBM Corp. Released 2010. IBM SPSS Statistics for Windows, Version 19.0. Armonk, New York).

## Results

**FIGURE 1:**
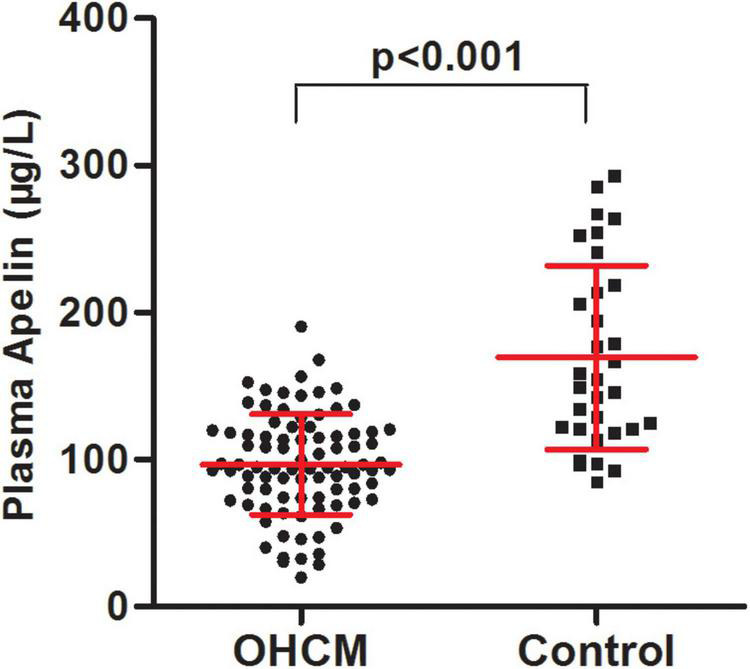
The patients with obstructive hypertrophic cardiomyopathy had lower plasma apelin levels in comparison with control subjects.

Clinical and echocardiographic characteristics of the study population are presented in Table 1. There were 88 patients with OHCM and 32 control subjects. The mean age of OHCM patients was 48.8 ± 13.1 years, and 57 (65%) of them were male. Plasma apelin levels were significantly lower in patients with OHCM than those in control subjects (96.6 ± 34.3 vs. 169.4 ± 62.5 μg/L, *p* < 0.001; Figure 1). Twenty-three (24%) of patients with OHCM had hypertension and 7 (8%) had diabetes. β-Blockers were taken in 64 (74%) patients, and calcium channel blockers in 26 (30%).

**TABLE 1:** Baseline characteristics of patients with OHCM and control subjects.

Variable	Patients with OHCM (*n* = 88)	Control subjects (*n* = 32)	*P-value*
Apelin (μg/L)	96.6 ± 34.3	169.4 ± 62.5	<0.001
Age (years)	48.8 ± 13.1	46.8 ± 5.9	0.262
Male, *n* (%)	57 (65%)	21 (66%)	1.000
BMI (kg/m^2^)	25.9 ± 3.4	26.9 ± 5.8	0.230
Systolic blood pressure, mmHg	120.6 ± 15.9	116.9 ± 11.7	0.240
Diastolic blood pressure, mmHg	74.0 ± 9.8	75.3 ± 8.5	0.508
Heart rate, beats/min	72.6 ± 10.8	67.9 ± 6.7	0.013
Dyspnea, *n* (%)	71 (81%)	0 (0%)	<0.001
Atrial fibrillation, *n* (%)	15 (17%)	1 (3%)	0.066
Hypertension, *n* (%)	21 (24%)	0 (0%)	0.001
Hyperlipidemia, *n* (%)	35 (40%)	13 (41%)	1.000
Diabetes mellitus, *n* (%)	7 (8%)	0 (0%)	0.187
Current smokers, *n* (%)	26 (30%)	8 (25%)	0.819
**Medications, *n* (%)**			
β-Blockers	64 (74%)	0 (0%)	<0.001
Calcium channel blockers	26 (30%)	0 (0%)	<0.001
ACEI/ARB	8 (9%)	0 (0%)	0.106
Statins	15 (17%)	0 (0%)	0.011
Diuretics	5 (6%)	0 (0%)	0.323
**Echocardiography**			
Septal wall thickness (mm)	22.9 ± 5.2	8.6 ± 0.9	<0.001
Left atrium diameter (mm)	41.1 ± 6.5	32.6 ± 3.0	<0.001
LVend-diastolic diameter (mm)	42.2 ± 5.2	47.4 ± 3.5	<0.001
LVejection fraction (%)	69.0 ± 4.8	65.3 ± 3.4	<0.001
Systolic anterior motion	82 (93%)	0 (0%)	<0.001
LVOTG at rest (mmHg)	79.1 ± 39.3	4.8 ± 0.6	<0.001
Mitral regurgitation*	62 (72%)	0 (0%)	<0.001
NT-proBNP (pmol/L)	1001.0 (435.6–2150.5)	19.8 (13.4–41.4)	<0.001

*ACEI, angiotensin-converting enzyme inhibitor; ARB, angiotensin receptor blocker; BMI, body mass index; OHCM, obstructive hypertrophic cardiomyopathy; LV, left ventricular; LVOTG, LV outflow tract gradient; NT-proBNP, N-terminal pro-B-type natriuretic peptide.*

*Moderate to severe mitral regurgitation. Data are expressed as mean ± SD, number (percentage), or median (interquartile range).

Echocardiographic data showed that patients had significantly greater septal wall thickness (22.7 ± 5.2 vs. 8.6 ± 0.9 mm, *p* < 0.001). The LVOT gradient at rest was 78.5 ± 39.1 mmHg. These data indicated the patients suffered from severe left ventricular hypertrophy and LVOT obstruction, whereas control subjects did not have cardiac abnormity.

Table 2 shows characteristics of patients with OHCM according to plasma apelin levels stratified by mean value. Patients with lower apelin levels (plasma apelin ≤ 96.6 μg/L) had greater septal wall thickness than those with higher apelin levels (plasma apelin >96.6 μg/L; 25.6 ± 5.5 vs. 23.2 ± 4.3 mm, *p* = 0.035). In contrast, the right ventricular end-diastolic diameter was greater in patients with higher apelin levels (23.0 ± 3.6 vs. 20.4 ± 3.3 mm, *p* = 0.001). Besides, there were marginally more patients suffering from heart failure of NYHA functional class III or IV in lower apelin group (33 vs. 15%, *p* = 0.053). Interestingly, the apelin levels of patients group with higher apelin were still lower than those of control subjects (125.4 ± 20.4 vs. 169.4 ± 62.5 μg/L, *p* < 0.001; Supplementary Figure 1).

**TABLE 2:** Characteristics of patients with obstructive hypertrophic cardiomyopathy according to plasma apelin levels stratified by mean value.

Variable	Plasma apelin (μ g/L)		*P-value*
	≤96.6 (*n* = 48) > 96.6 (*n* = 40)		
Age (years)	49.0 ± 12.8	48.5 ± 13.6	0.835
Male, *n* (%)	31 (65%)	26 (65%)	1.000
BMI (kg/m^2^)	25.5 ± 3.7	26.4 ± 2.9	0.215
Systolic blood pressure (mmHg)	119.9 ± 16.7	121.5 ± 15.1	0.660
Diastolic blood pressure (mmHg)	73.7 ± 9.9	74.4 ± 9.7	0.769
Heart rate (beats/min)	73.2 ± 8.1	71.9 ± 13.4	0.597
NYHA functional class III or IV, n (%)	16 (33%)	6 (15%)	0.053
Chest pain, *n* (%)	20 (42%)	17 (43%)	1.000
Palpitation, *n* (%)	16 (33%)	9 (23%)	0.344
Family history of HCM, *n* (%)	3 (6%)	5 (13%)	0.458
Atrial fibrillation, *n* (%)	9 (19%)	6 (15%)	0.778
**Cardiovascular risk, *n* (%)**			
Hypertension	12 (25%)	9 (23%)	0.808
Diabetes mellitus	6 (13%)	1 (3%)	0.121
Hyperlipidemia	17 (35%)	18 (45%)	0.389
Current smokers	17 (35%)	9 (23%)	0.242
**Medications, *n* (%)**			
β-Blockers	34 (74%)	30 (75%)	1.000
Calcium channel blockers	14 (29%)	12 (31%)	1.000
ACEI/ARB	6 (13%)	2 (5%)	0.293
Statins	8 (17%)	7 (18%)	1.000
Diuretics	4 (8%)	1 (3%)	0.371
Trimetazidine	3 (6%)	2 (5%)	1.000
NT-proBNP (pmol/L)	1488.5 (798.0–3303.5)	1151.2 (678.0–2202.6)	0.156
LVOTG at rest (mmHg)	72.5 (53.3–112.0)	81.0 (58.0–107.0)	0.640
**CMR imaging**			
Septal wall thickness (mm)	25.6 ± 5.5	23.2 ± 4.3	0.035
RV end-diastolic diameter (mm)	20.4 ± 3.3	23.0 ± 3.6	0.001
Left atrium diameter (mm)	43.4 ± 7.8	42.5 ± 6.7	0.582
Left atrium volume (ml)	115.8 ± 43.6	122.2 ± 42.3	0.497
Left atrium volume index (ml/m^2^)	65.8 ± 24.7	66.7 ± 22.7	0.863
LV end-diastolic diameter (mm)	45.7 ± 4.6	45.4 ± 6.1	0.819
LV end-diastolic volume (ml)	135.6 ± 34.3	137.3 ± 27.5	0.803
LV end-diastolic volume index (ml/m^2^)	75.9 ± 17.8	75.1 ± 15.0	0.825
LV mass (g)	163.6 ± 73.2	153.7 ± 53.2	0.492
LV mass index (g/m^2^)	92.5 ± 41.2	85.1 ± 30.7	0.372
LV ejection fraction (%)	64.6 ± 9.5	65.3 ± 8.0	0.709
Cardiac index (L/min/m^2^)	3.3 ± 1.0	3.3 ± 0.8	0.862

*CK-MB, creatine kinase MB; CMR, cardiovascular magnetic resonance; NYHA, New York Heart Association; RV, right ventricular; other abbreviations as in Table 1. Data are expressed as mean ± SD, number (percentage), or median (interquartile range).*

The plasma apelin levels with respect to presence or not of clinical characteristics in the patients with OHCM were depicted in Supplementary Table 1. There were no significant differences in the plasma apelin levels between OHCM patients with hypertension, diabetes mellitus, hyperlipidemia, and patients without these cardiovascular risk factors.

**FIGURE 2:**
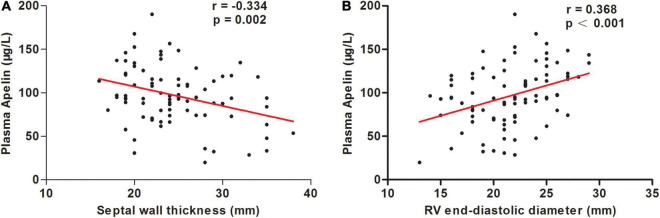
Correlations between plasma apelin levels and septal wall thickness **(A)** and right ventricular (RV) end-diastolic diameter **(B)** in patients with obstructive hypertrophic cardiomyopathy.

The correlations between clinical characteristics and apelin levels in the patients with OHCM are shown in Table 3. Plasma apelin levels were inversely correlated with septal wall thickness (SWT; *r* = −0.334, *p* = 0.002; Figure 2A) and Ln (NT-proBNP) (*r* = −0.307, *p* = 0.008). In contrast, plasma apelin levels were positively correlated with right ventricular end-diastolic diameter (RVEDD; *r* = 0.368, *p* < 0.001; Figure 2B) and body mass index (BMI; *r* = 0.287, *p* = 0.008). In multiple linear regression analysis, SWT was independently associated with decreasing apelin values, whereas RVEDD was independently associated with increasing apelin values (Table 4).

**TABLE 3:** Correlation between clinical characteristics and plasma apelin levels of patients with obstructive hypertrophic cardiomyopathy.

Variable	Correlation coefficient (*r*)	*P-value*
Septal wall thickness (mm)	–0.334	0.002
RV end-diastolic diameter (mm)	0.368	<0.001
BMI (kg/m^2^)	0.287	0.008
Ln (NT-proBNP)	–0.307	0.008

*Abbreviations as in Tables 1, 2.*

**TABLE 4:** Multiple linear regression analysis for the association between plasma apelin levels and clinical characteristics in patients with obstructive hypertrophic cardiomyopathy.

Variable	Standardized coefficients (β)	*P-value*
Septal wall thickness (mm)	−0.274	0.018
RV end-diastolic diameter (mm)	0.340	0.004

*Abbreviations as in Tables 1, 2. Multiple R = 0.456, R^2^ = 0.208.*

## Discussion

Although recent studies have unraveled that apelin may have multiple protective effects against development of several cardiovascular diseases (4, 11), there are few data on the plasma apelin levels in patients with OHCM or potential relations between apelin levels and clinical variables that reflect severity of the disease. For the first time, the present study revealed that plasma apelin levels were decreased in patients with OHCM. Furthermore, the septal wall thickness (SWT) was independently and inversely associated with plasma apelin levels, while the right ventricular end-diastolic diameter (RVEDD) was positively associated with plasma apelin levels.

HCM, particularly OHCM, is the most common inherited cardiomyopathy and the most common cause of sudden death in young people. So far, it is not difficult to diagnose OHCM in patients with obvious clinical symptoms using echocardiography and cardiac magnetic resonance imaging. Previous studies showed that existing biomarkers of cardiovascular diseases, such as cardiac troponin and N-terminal proB-type natriuretic peptide (NT-proBNP), could be employed to assess the severity of OHCM in terms of myocardial damage and heart failure (12). With regard to medical treatment, beta-blockers and non-dihydropyridine calcium channel blockers have long been “first line” pharmacotherapy in OHCM (13). Recently, mavacamten, a myosin adenosine triphosphatase (ATPase) inhibitor, markedly reduced post-exercise LVOT gradient and gave rise to symptoms improvement in patients with OHCM (14). However, mavacamten also showed several adverse effects. For instance, it led to too much decrease of LVEF and stress cardiomyopathy in some patients within 30 weeks (14). There might be more patients suffering dramatic LVEF reduction and stress cardiomyopathy if mavacamten is taken for a long time in a large population. Hence, there is still a great need to comprehensively identify the mechanism of OHCM so that optimal therapeutic measures may be found.

Since its discovery in 1998, apelin has attracted intense interest (15). Apelin is highly expressed in the heart and plays an important role in the regulation of cardiovascular functions, including inotrope, vasodilator, and diuretic (5). It has been reported that plasma apelin levels are decreased in several cardiovascular diseases. Our previous work revealed that plasma apelin concentration was reduced following acute coronary syndrome (ACS) and remained low to 6 months (16). This reduction of apelin may be in part due to elevated filling pressure in the LV after ACS (16, 17). Chong et al. investigated 202 patients with chronic heart failure secondary to LV systolic dysfunction and 22 control subjects, and observed that plasma apelin concentrations were decreased in patients (18). Furthermore, Chandrasekaran et al. reported similar observations and they found decrease of plasma apelin was owing to a reduction of myocardial apelin production (19). Considering the positive inotropic and vasodilative effects of apelin, the down-regulation may be an upstream event of heart failure. A variety of hypotheses have been proposed to explain why apelin levels are reduced in heart failure. The major mechanism may be that apelin is down-regulated by excessively activated renin–angiotensin system (11). Our data showed that plasma apelin levels were significantly decreased in patients with OHCM compared with control subjects. The mechanism that leads to reduction of apelin in patients with OHCM may be similar to that in heart failure with reduced LVEF, or due to its unique pathological changes of myocardium. Based on cardio-protective actions of apelin, potential benefits of apelin in treating acute heart failure have been assessed. Evidence from animal study indicated that exogenous administration of apelin improved LV systolic function in dogs with advanced heart failure (20). Additionally, Japp et al. performed a study in 18 patients with heart failure and 26 control subjects, and found that acute apelin administration in humans causes peripheral and coronary vasodilatation and increases cardiac output (21).

Several studies have suggested that apelin is involved in myocardial hypertrophy (1). Szokodi et al. reported that apelin gene expression was markedly down-regulated in cultured neonatal rat ventricular myocytes subjected to mechanical stretch and *in vivo* in two models of ventricular hypertrophy (17). Furthermore, apelin was found to ameliorate high fat diet-induced cardiac hypertrophy (22). In addition, our previous data showed that apelin could inhibit myocardial hypertrophy induced by angiotensin II in neonatal rat ventricular cardiomyocyte (7). Another study performed in 232 hypertensive patients without concomitant diseases affecting cardiovascular functions indicated that plasma apelin levels in hypertensive patients were significantly lower than those in controls (23). And previous studies surmised that atrium produces most of apelin in the heart (4, 11). In the present study, we found that plasma apelin levels were inversely associated with septal wall thickness (SWT) in patients with OHCM. By contrast, the LV mass was not independently associated with apelin levels. Therefore, our novel findings indicated that septal wall, rather than other segments of LV, is a determinant of plasma apelin in patients with OHCM. A study with human plasma and heart tissues by Foldes et al. raised the possibility that apelin may be produced predominantly in the atria (24). However, Foldes et al. determined apelin-36 levels, while our kit can detect all active forms of apelin, including apelin-36, apelin-31, apelin-28, and apelin 13. Apelin fragments other than apelin-36 might be produced predominantly in the septal wall. The pathological septal hypertrophy might retard apelin expression, or otherwise, reduction of apelin promotes pathogenesis of septal hypertrophy in OHCM. Further experiment is necessary to elucidate how apelin is reduced in OHCM.

Hitherto, there were scarce data regarding the role of apelin in right ventricle (RV). In a recent study concerning right ventricular function in pulmonary hypertension, Frump et al. demonstrated that apelin are also expressed in RV (25). Apelin was decreased in RV failure but not in adaptive RV remodeling, indicating protective effects of apelin against RV failure development (25). In the current study, we observed that plasma apelin was positively associated with right ventricular end-diastolic diameter (RVEDD) in patients with OHCM. Given that the OHCM patients in our study did not have RV failure, the change of apelin in relation to RVEDD may be an adaptive regulation to improve RV function. Thus, our data also suggested beneficial effects of apelin in RV. Further work is necessary to investigate the role of apelin in structural and functional remodeling of RV.

This study has some limitations that warrant discussion. First, lower plasma apelin levels in patients with OHCM compared with those in control subjects may be confounded by hypertension or diabetes, which were only in patients group. However, further analysis showed that the presence of hypertension and diabetes or not did not affect apelin levels in patients with OHCM. Second, the study population all had left ventricular outflow tract (LVOT) obstruction. Therefore, the findings of this study do not apply to patients without LVOT obstruction. Additionally, this is a cross-sectional study, which renders conclusions about the causality of demonstrated relations impossible.

## Conclusion

Plasma apelin levels are reduced in patients with OHCM. The apelin levels are inversely related to SWT and positively related to RVEDD.

## Data Availability Statement

The raw data supporting the conclusions of this article will be made available by the authors, without undue reservation.

## Ethics Statement

The studies involving human participants were reviewed and approved by the Ethics Committee of Fuwai Hospital (Beijing, China). The patients/participants provided their written informed consent to participate in this study.

## Author Contributions

ZJ, JY, and SQ contributed to the conception and design. CY, CZ, and RJ contributed to the manuscript writing. CY, CZ, JY, and SQ contributed to the provision of study materials or participants, collection, and assembly of data. All authors contributed to the data analysis and interpretation, and approval of final version of the manuscript.

## Conflict of Interest

The authors declare that the research was conducted in the absence of any commercial or financial relationships that could be construed as a potential conflict of interest.

## Publisher’s Note

All claims expressed in this article are solely those of the authors and do not necessarily represent those of their affiliated organizations, or those of the publisher, the editors and the reviewers. Any product that may be evaluated in this article, or claim that may be made by its manufacturer, is not guaranteed or endorsed by the publisher.

## References

[1] Usaj M Moretto L Mansson A. Critical evaluation of current hypotheses for the pathogenesis of hypertrophic cardiomyopathy. *Int J Mol Sci.* (2022) 23:2195. 10.3390/ijms23042195 35216312 PMC8880276

[2] Maron BJ Desai MY Nishimura RA Spirito P Rakowski H Towbin JA Diagnosis and evaluation of hypertrophic cardiomyopathy: JACC State-of-the-Art review. *J Am Coll Cardiol.* (2022) 79:372–89. 10.1016/j.jacc.2021.12.002 35086660

[3] Tatemoto K Hosoya M Habata Y Fujii R Kakegawa T Zou MX Isolation and characterization of a novel endogenous peptide ligand for the human APJ receptor. *Biochem Biophys Res Commun.* (1998) 251:471–6. 10.1006/bbrc.1998.9489 9792798

[4] Liu W Yan J Pan W Tang M. Apelin/Elabela-APJ: a novel therapeutic target in the cardiovascular system. *Ann Transl Med.* (2020) 8:243. 10.21037/atm.2020.02.07 32309390 PMC7154429

[5] Folino A Montarolo PG Samaja M Rastaldo R. Effects of apelin on the cardiovascular system. *Heart Fail Rev.* (2015) 20:505–18. 10.1007/s10741-015-9475-x 25652330

[6] Scimia MC Hurtado C Ray S Metzler S Wei K Wang J APJ acts as a dual receptor in cardiac hypertrophy. *Nature.* (2012) 488:394–8. 10.1038/nature11263 22810587 PMC3422434

[7] Zhou Y Chen Y Qiao S. Effect of Apelin on angiotensin II-induced cardiomyocyte hypertrophy with its mechanism in experimental rats. *Chin Circ J.* (2014) 29:733–7.

[8] Elliott PM Anastasakis A Borger MA Borggrefe M Cecchi F Charron P 2014 ESC Guidelines on diagnosis and management of hypertrophic cardiomyopathy: the task force for the diagnosis and management of hypertrophic cardiomyopathy of the European Society of Cardiology (ESC). *Eur Heart J.* (2014) 35:2733–79. 10.1093/eurheartj/ehu284 25173338

[9] Nagueh SF Bierig SM Budoff MJ Desai M Dilsizian V Eidem B American Society of Echocardiography clinical recommendations for multimodality cardiovascular imaging of patients with hypertrophic cardiomyopathy: endorsed by the American Society of Nuclear Cardiology, Society for Cardiovascular Magnetic Resonance, and Society of Cardiovascular Computed Tomography. *J Am Soc Echocardiogr.* (2011) 24:473–98. 10.1016/j.echo.2011.03.006 21514501

[10] Yang C Zhang C Yuan J Cui J Qiao S. Prevalence and determinants of elevated D-dimer in patients with hypertrophic cardiomyopathy. *Biomark Med.* (2020) 14:131–40. 10.2217/bmm-2019-0225 32057272

[11] Goidescu CM Vida-Simiti LA. The Apelin-APJ system in the evolution of heart failure. *Clujul Med.* (2015) 88:3–8. 10.15386/cjmed-380 26528040 PMC4508609

[12] Zhang C Liu R Yuan J Cui J Hu F Yang W Significance and determinants of cardiac troponin i in patients with obstructive hypertrophic cardiomyopathy. *Am J Cardiol.* (2015) 116:1744–51. 10.1016/j.amjcard.2015.09.006 26434514

[13] Maron BJ Desai MY Nishimura RA Spirito P Rakowski H Towbin JA Management of hypertrophic cardiomyopathy: JACC State-of-the-Art review. *J Am Coll Cardiol.* (2022) 79:390–414. 10.1016/j.jacc.2021.11.021 35086661

[14] Spertus JA Fine JT Elliott P Ho CY Olivotto I Saberi S Mavacamten for treatment of symptomatic obstructive hypertrophic cardiomyopathy (EXPLORER-HCM): health status analysis of a randomised, double-blind, placebo-controlled, phase 3 trial. *Lancet.* (2021) 397:2467–75. 10.1016/S0140-6736(21)00763-7 34004177

[15] Yu XH Tang ZB Liu LJ Qian H Tang SL Zhang DW Apelin and its receptor APJ in cardiovascular diseases. *Clin Chim Acta.* (2014) 428:1–8. 10.1016/j.cca.2013.09.001 24055369

[16] Zhou Y Wang Y Qiao S. Apelin: a potential marker of coronary artery stenosis and atherosclerotic plaque stability in ACS patients. *Int Heart J.* (2014) 55:204–12. 10.1536/ihj.13-234 24806385

[17] Szokodi I Tavi P Foldes G Voutilainen-Myllyla S Ilves M Tokola H Apelin, the novel endogenous ligand of the orphan receptor APJ, regulates cardiac contractility. *Circ Res.* (2002) 91:434–40. 10.1161/01.res.0000033522.37861.69 12215493

[18] Chong KS Gardner RS Morton JJ Ashley EA McDonagh TA. Plasma concentrations of the novel peptide apelin are decreased in patients with chronic heart failure. *Eur J Heart Fail.* (2006) 8:355–60. 10.1016/j.ejheart.2005.10.007 16464638

[19] Chandrasekaran B Kalra PR Donovan J Hooper J Clague JR McDonagh TA. Myocardial apelin production is reduced in humans with left ventricular systolic dysfunction. *J Card Fail.* (2010) 16:556–61. 10.1016/j.cardfail.2010.02.004 20610231

[20] Wang M Gupta RC Rastogi S Kohli S Sabbah MS Zhang K Effects of acute intravenous infusion of apelin on left ventricular function in dogs with advanced heart failure. *J Card Fail.* (2013) 19:509–16. 10.1016/j.cardfail.2013.05.004 23834927 PMC3706995

[21] Japp AG Cruden NL Barnes G van Gemeren N Mathews J Adamson J Acute cardiovascular effects of apelin in humans: potential role in patients with chronic heart failure. *Circulation.* (2010) 121:1818–27. 10.1161/CIRCULATIONAHA.109.911339 20385929

[22] Ceylan-Isik AF Kandadi MR Xu X Hua Y Chicco AJ Ren J Apelin administration ameliorates high fat diet-induced cardiac hypertrophy and contractile dysfunction. *J Mol Cell Cardiol.* (2013) 63:4–13. 10.1016/j.yjmcc.2013.07.002 23859766

[23] Przewlocka-Kosmala M Kotwica T Mysiak A Kosmala W. Reduced circulating apelin in essential hypertension and its association with cardiac dysfunction. *J Hypertens.* (2011) 29:971–9. 10.1097/HJH.0b013e328344da76 21346619

[24] Foldes G Horkay F Szokodi I Vuolteenaho O Ilves M Lindstedt KA Circulating and cardiac levels of apelin, the novel ligand of the orphan receptor APJ, in patients with heart failure. *Biochem Biophys Res Commun.* (2003) 308:480–5. 10.1016/s0006-291x(03)01424-4 12914775

[25] Frump AL Albrecht M Yakubov B Breuils-Bonnet S Nadeau V Tremblay E 17Beta-Estradiol and estrogen receptor alpha protect right ventricular function in pulmonary hypertension via BMPR2 and apelin. *J Clin Invest.* (2021) 131:e129433. 10.1172/JCI129433 33497359 PMC7968046

