# Supplementary figures and images for: Significance and Determinants of Plasma Apelin in Patients With Obstructive Hypertrophic Cardiomyopathy
Source: Front Cardiovasc Med. 2022 Jun 17;9:904892. doi: 10.3389/fcvm.2022.904892 (PMC9247182; doi:10.3389/fcvm.2022.904892)

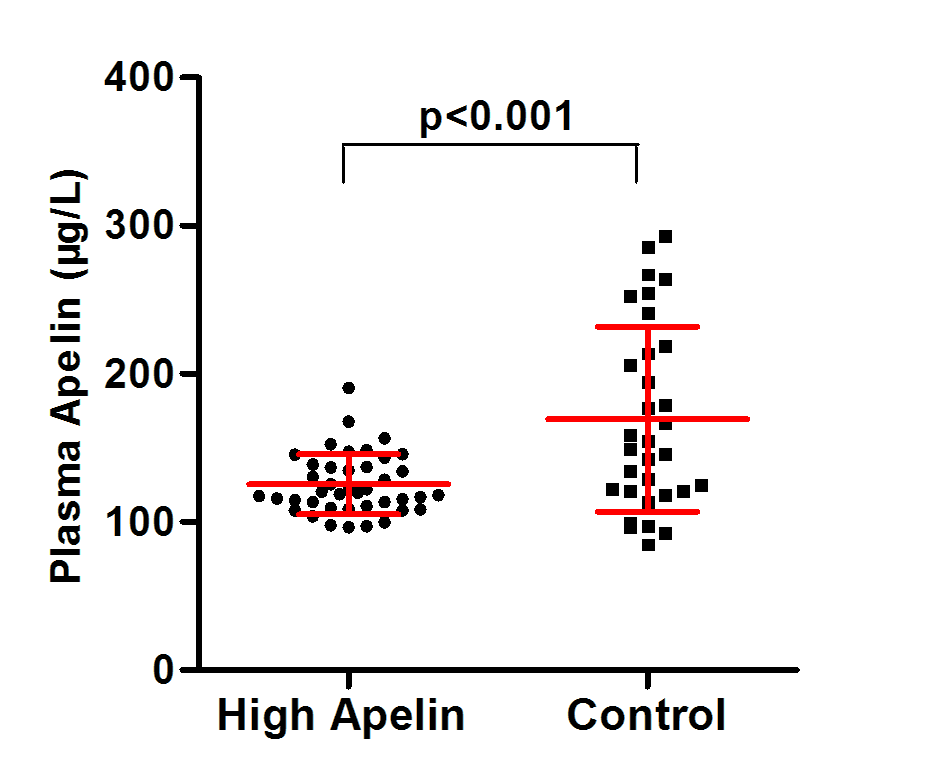

Supplement: Supplementary file 1 [file Image_1.TIF]
