# Supplementary material for: Significance and Determinants of Plasma Apelin in Patients With Obstructive Hypertrophic Cardiomyopathy
Source: Front Cardiovasc Med. 2022 Jun 17;9:904892. doi: 10.3389/fcvm.2022.904892 (PMC9247182; doi:10.3389/fcvm.2022.904892)
Supplement: Supplementary file 2 [file Table_1.DOCX]

***Supplementary Material***

**Supplemental Table 1.** The plasma apelin levels according to clinical variables of patients with obstructive hypertrophic cardiomyopathy.

| **Variable** | **Plasma apelin (μg/L)** | | ***p* Value** |
| --- | --- | --- | --- |
|  | **Presence of variables** | **Absence of variables** |  |
| Male | 95.5 ± 33.3 | 98.6 ± 36.5 | 0.688 |
| NYHA functional class Ⅲ or Ⅳ | 87.1 ± 28.2 | 99.8 ± 35.7 | 0.131 |
| Chest pain | 98.1 ± 36.5 | 95.5 ± 32.9 | 0.730 |
| Palpitation | 90.4 ± 45.4 | 99.1 ± 28.8 | 0.284 |
| Syncope | 101.4 ± 37.1 | 95.5 ± 33.7 | 0.526 |
| Family history of HCM | 116.6 ± 46.7 | 94.6 ± 32.7 | 0.085 |
| Atrial fibrillation | 97.3 ± 39.4 | 96.5 ± 33.4 | 0.935 |
| Hypertension | 102.9 ± 30.5 | 94.7 ± 35.4 | 0.340 |
| Diabetes mellitus | 89.2 ± 18.5 | 97.3 ± 35.3 | 0.551 |
| Hyperlipidaemia | 105.1 ± 34.6 | 91.0 ± 33.1 | 0.058 |
| Current smokers | 93.1 ± 36.3 | 98.1 ± 33.6 | 0.535 |
| β-Blockers | 95.4 ± 37.4 | 101.2 ± 25.3 | 0.417 |
| Calcium channel blockers | 94.3 ± 32.4 | 96.8 ± 35.0 | 0.759 |
| ACEI/ARB | 88.8 ± 31.2 | 96.2 ± 33.5 | 0.550 |
| Statins | 102.4 ± 40.5 | 95.4 ± 33.0 | 0.477 |
| Diuretics | 72.6 ± 24.1 | 98.1 ± 34.4 | 0.107 |
| Trimetazidine | 106.9 ± 26.6 | 96.0 ± 34.7 | 0.495 |

ACEI, angiotensin-converting enzyme inhibitor; ARB, angiotensin receptor blocker; HCM, hypertrophic cardiomyopathy; NYHA, New York Heart Association.

Data are expressed as mean ± SD.

**Figure caption:**

**Supplemental Figure S1.** When the patients with obstructive hypertrophic cardiomyopathy (OHCM) were divided into two groups according to mean value of plasma apelin levels, the apelin levels of patients group with higher apelin were still lower than those of control subjects.
